# Transcriptomic profiling of wheat stem during meiosis in response to freezing stress

**DOI:** 10.3389/fpls.2022.1099677

**Published:** 2023-01-12

**Authors:** Danyu Yao, Juan Wang, Wentao Peng, Bowen Zhang, Xiaolan Wen, Xiaoneng Wan, Xiuyuan Wang, Xinchun Li, Jian Ma, Xiaofen Liu, Yinglun Fan, Guozhong Sun

**Affiliations:** ^1^ National Engineering Laboratory of Crop Molecular Breeding, Institute of Crop Science, Chinese Academy of Agricultural Sciences, Beijing, China; ^2^ College of Agricultural Science and Engineering, Liaocheng University, Liaocheng, Shandong, China; ^3^ College of Agronomy, Jilin Agricultural University, Changchun, Jilin, China; ^4^ College of Landscape and Ecological Engineering, Hebei University of Engineering, Handan, Hebei, China

**Keywords:** *Triticum aestivum*, spring frost injury, transcriptome, hormone signal transduction, transcription factor, cell wall

## Abstract

Low temperature injury in spring has seriously destabilized the production and grain quality of common wheat. However, the molecular mechanisms underlying spring frost tolerance remain elusive. In this study, we investigated the response of a frost-tolerant wheat variety Zhongmai8444 to freezing stress at the meiotic stage. Transcriptome profiles over a time course were subsequently generated by high-throughput sequencing. Our results revealed that the prolonged freezing temperature led to the significant reductions in plant height and seed setting rate. Cell wall thickening in the vascular tissue was also observed in the stems. RNA-seq analyses demonstrated the identification of 1010 up-regulated and 230 down-regulated genes shared by all time points of freezing treatment. Enrichment analysis revealed that gene activity related to hormone signal transduction and cell wall biosynthesis was significantly modulated under freezing. In addition, among the identified differentially expressed genes, 111 transcription factors belonging to multiple gene families exhibited dynamic expression pattern. This study provided valuable gene resources beneficial for the breeding of wheat varieties with improved spring frost tolerance.

## Introduction

Wheat (*Triticum aestivum* L.) is one of the most widely grown staple crops worldwide. The spring frost injury has become the limiting factor of wheat production because it has dramatically influenced the yield, quality, and the geographical distribution of wheat (Chen et al., 2020). The Yellow and Huai River Valley Winter Wheat Zone (YHVWWZ) is the major producing area of China’s winter wheat. Frost damage in spring generally occurs from February to April, when the plants are at the jointing to the booting stage of wheat growth. At this time, the wheat tissues are tender and contain a lot of water, so they are easy to get damaged (Frederiks et al., 2015). Exposure to low temperature may cause the damage of leaf, stem and young spike, which could result in the decrease in the grain number and quality. From 2001 to 2021, the frequency of spring frost injury in the YHVWWZ was as high as 40% (Liu et al., 2021). In 2018, it has caused the yield losses of 3,000 kg per hectare in wheat varieties with weak freezing tolerance when compared to freezing-resistant varieties (Ou and Wang, 2019). Therefore, breeding and promoting freezing-tolerant wheat varieties is an important approach to deal with the disaster of spring frost injury in YHVWWZ. However, at present, knowledge of the physiological mechanisms underlying the freezing tolerance is still elusive, limiting the genetic improvement of wheat varieties.

Low temperature stress includes both cold/chilling stress (>0°C) and freezing stress (<0°C). When exposed to low temperature, plants can perceive the signal and induce a series of complex changes in their morphological structure, internal substances and photosynthesis (Ding et al., 2019). The accumulation of soluble sugar and amino acid was observed in many plant species (Nakabayashi and Saito, 2015). A series of mechanisms are initiated to protect and maintain the normal metabolism and growth of plants, so as to avoid or minimize the damage of low temperature. During this complex biological process, a large number of genes are activated and play an important role for plants to survive. For instance, transcription factors (TFs) are thought to control many cold responsive genes through direct binding to cis-acting elements in the promoter regions. Regulation of cold-regulated (*COR*) genes by CRT/DRE-binding factors (CBFs) constitutes the predominant cold signaling pathway and was found to be conserved in both dicots and monocots (Park et al., 2015). In diploid *Triticum monococcum*, three *CBF* genes (*CBF12, CBF14, CBF15*) have been identified to be highly associated with frost tolerance (Knox et al., 2008). Overexpression of *CBF14* and *CBF15* from winter wheat resulted in the enhanced frost tolerance in spring barley (Soltész et al., 2013). The CBF transcription factors belong to the APETALA2/Ethylene response element binding protein (AP2/EREBP) family. Other transcription factors including MYB, NAC, bZIP, and WRKY TFs have also been identified to be linked to the tolerance of low temperature stress (Su et al., 2010; Mao et al., 2012).

In addition, phytohormone always acts as a crucial signal to modulate multiple plant processes and has been reported to play an essential role for plant to cope with low temperature stress. A key hormone is abscisic acid (ABA), which not only controls the internal water deficiency by regulating stomatal conductance but also regulates the transcriptome of downstream stress-related genes. It has been shown that CBF transcription factors induced the biosynthesis of ABA, which subsequently stimulated the expression of *COR* genes (Sharabi-Schwager et al., 2010; Wang et al., 2016). The exogenous application of ABA significantly improved the low-temperature tolerance of tomato (Kim et al., 2002), pepper (Guo et al., 2012), magnolia liliiflora (Yang et al., 2016) and wheat (Yu et al., 2020). Moreover, accumulating evidence indicated that jasmonic acid (JA) could mediate the responses to low temperature stress with other hormones, such as ABA, auxin, ethylene, and gibberellin (Hu et al., 2017). Wang et al. (2016) reported that JA could activate the ABA-dependent CBF signaling pathway and positively modulate the reactions to cold stress in tomato. Blocking of JA biosynthesis and signaling caused the hypersensitivity to freezing stress (Hu et al., 2013). In Arabidopsis, it has been reported that ethylene and gibberellin (GA) negatively regulated plant cold tolerance (Shi et al., 2012; Richter et al., 2013). For instance, a GA-deficient mutant showed increased expression level of cold-responsive genes and enhanced resistance to cold stress (Richter et al., 2013). These studies indicate the complex nature of plant’s responses to low temperature.

It has been suggested that properties of cell wall strongly influence the frost resistance of plants. When subjected to freezing, ice crystallization takes place in the intercellular space which could lead to the dehydration and mechanical damage of cells. Plasma membrane and cell wall work together to perceive the external signal of freezing stress and in turn cause the corresponding changes in cell wall content, composition and structure. This phenomenon has been observed in multiple plant species such as Arabidopsis, winter wheat, maize and tobacco (Sugiyama and Shimazaki, 2007; Bilska-Kos et al., 2016; Willick et al., 2017; Parrotta et al., 2019; Takahashi et al., 2019). There is increasing evidence showing that cell wall genes were involved in the regulation of plant low temperature tolerance. A transcriptomic study performed by Le et al. (2015) showed that several cell wall genes were dramatically up regulated in Arabidopsis accessing to sub-zero temperatures. Mutant lacking *XTH19* gene, which encode a xyloglucan endotransglucosylase/hydrolase, showed hypersensitivity to low-temperature stress compared to wild-type plants (Takahashi et al., 2021). In addition, a novel frost tolerant gene, *SENSITIVE-TO-FREEZING8* (*SFR8*), has been cloned and demonstrated to influence pectin fucosylation (Panter et al., 2019).

Large-scale analysis of gene expression pattern by transcriptome techniques provides a cost-effective strategy for elucidating the molecular mechanisms underlying physiological processes and it can substantially increase the efficiency of identifying genes of interest. A range of plant species such as Arabidopsis, rice, wheat, maize, and cotton have been extensively subjected to transcriptional programming in response to different stresses (Jian et al., 2014; Huang et al., 2019; Sga et al., 2019; Wang et al., 2019). Zhao et al. (2019) integrated transcriptome and metabolome analyses for understanding how wheat plants respond to low temperature. 29,066 DEGs were identified, and pathways of phytohormone signaling and proline biosynthesis were shown to be significantly modulated under freezing treatment.

The objective of this study is to investigate the phenotypic changes as well as transcriptome dynamics of wheat cultivar zhongmai8444 in response to freezing temperature at the meiosis stage. Our results will provide valuable insights into understanding molecular mechanisms underlying the frost tolerance and help identify new gene resource that is beneficial for genetic improvement of spring frost tolerance in wheat.

## Materials and methods

### Plant materials, freezing treatment, and seed setting measurement

Zhongmai8444 is a spring wheat variety breed in our lab. It showed great freezing tolerance since it could survive in the winter of Beijing (-5°C to 2°C) by field trials for five consecutive years. Zhongmai8444 was grown in a growth chamber maintaining at 22°C during the day, 15°C during the night, 16-h light, 8-h dark, 80% humidity and a PPFD of 450 µmol m^-2^ s^-1^. Three plants were grown in each pot and their growth stages were recorded according to the Zadoks scale (Zadoks et al., 1974). Developmental stages of pollen mother cells from immature anthers were examined following Draeger and Moore (2017). When the distance from flag leaf to the adjacent leaf was around 2-3 cm (**Supplemental Figure S1**), Zhongmai8444 was verified to be at meiotic stage and subjected to freezing treatment shown in **Supplemental Figure S2**. Wheat plants grown at 22°C (normal temperature growth) prior to freezing were set as the control group (time point T1). Temperature in growth chamber was gradually decreased from 22°C to -2.5°C using 6 h (time point T2) and then maintained at -2.5°C for 48 h (time point T4). Plants were also harvested after frozen at −2.5°C for 24 h (time point T3). Finally, temperature gradually increased from -2.5°C to 22°C (time point T5) and plants were collected as recovery group. Samples were named as J1 to J5 according to the time points (T1 to T5). Stem tissues from six individual plants were pooled (with three replications) and immediately frozen in liquid nitrogen for subsequent RNA extraction. In addition, another set of plants were treated with freezing (-2.5°C for 48 h) during meiosis as described above and transferred to normal growth condition for continuing growth. Plants that were maintained at normal growth condition were used as the control. The seed setting rate was analyzed using the equation: seed setting rate=grain number each spike/flower number each spike × 100%.

### Cell wall thickness measurement

To investigate the cell wall morphology of stem tissue after freezing treatment, xylem cells from three individual plants were selected for further measurement. Thin cross-sections were cut from the base of wheat stem using a sterile razor blade and were subsequently incubated in 0.02% toluidine blue solution for 2 min at room temperature. After thoroughly washing, respective stem cross-sections were mounted in water prior to imaging on an OLYMPIC CX41RF microscope equipped with an EOS 450D camera (Canon). Cell wall morphology and thickness of the xylem cells were subsequently analyzed using the Image J software (National Institutes of Health; http://rsb.info.nih.gov/ij/). The cell wall thickness was analyzed from at least 20 xylem cells collected from three individual plants. For the observation by a transmission electron microscope (TEM), samples were prepared following Vigani et al. (2018) with some modifications. Briefly, stem tissues were fixed overnight in 3% (v/v) glutaraldehyde at room temperature and subsequently post fixed with 1% (w/v) osmium tetroxide for 2 h at 4°C. After dehydration in a graded ethanol series, samples were embedded in resin for ultrathin sectioning and visualization with a H-7500 TEM (HITACH, Japan) at 80 kV.

### RNA extraction and sequencing

Total RNA was extracted from stem tissues using an RNAprep Pure Plant Kit (Tiangen) according to the manufacturer’s instructions and its quantity, quality, and integrity were evaluated using an Agilent 2100 Bioanalyzer system (Agilent Technologies, Palo Alto, CA, USA). Purified RNA was sent to Novogene (https://cn.novogene.com/) for RNA sequencing using Illumina NovaSeq platform and 150 bp paired-end reads were generated. Clean reads were obtained by removing reads with adapters sequences, reads with poly-N and low-quality reads using in house scripts. Then, the reads after quality control were mapped to the wheat reference genome (https://urgi.versailles.inra.fr/download/iwgsc/IWGSC_RefSeq_Assemblies/v1.0/) using Hisat2 v2.0.5 (Kim et al., 2015). Wheat gene annotation was obtained from https://urgi.versailles.inra.fr/download/iwgsc/IWGSC_RefSeq_Annotations/v1.0/iwgsc_refseqv1.0_HighConf_2017Mar13.gff3.zip.

Gene expression levels were quantified as Fragments Per Kilobase of transcript sequence per million mapped fragments (FPKM) using the featureCounts v1.5.0-p3 program (Yang et al., 2014). Principal component analysis (PCA) was performed to evaluate the reproducibility of different samples under the same treatment.

### Differential expression analysis

Differential gene expression analysis was performed using the DESeq2 (Love et al., 2014) package in R (1.20.0). The Benjamini and Hochberg method was used to adjust P-values to control the false discovery rate (FDR). Differentially expressed genes were identified according to the threshold of |log2FoldChange| > 1 and FDR < 0.05. Gene Ontology (GO) and Kyoto Encyclopedia of Gene and Genomes (KEGG) pathways enrichment analysis of the DEGs were performed using clusterProfiler R package (Yu et al., 2012).

### Real-time quantitative PCR

Real-time quantitative PCR was performed on the stem tissues to examine expression patterns of selected genes. Samples were run in triplicate with SuperReal SYBR Green PreMix Plus (Tiangen) on a Bio-Rad CFX96 Touch Real-Time PCR Detection System. The PCR reactions were performed under the following conditions: 95°C for 15 min, followed by 40 cycles of 95°C for 10 s, 58°C for 20 s, and 72°C for 30 s. Relative quantities were calculated and normalized to the actin gene, which was used as an internal control, using the 2^-ΔΔCt^ algorithm. The data are shown as means ± standard errors (SE) and primers used for qRT-PCR analysis are listed in **Supplementary Table S4**.

## Results

### Phenotypic analysis of Zhongmai8444 after freezing treatment

**Figure 1:**
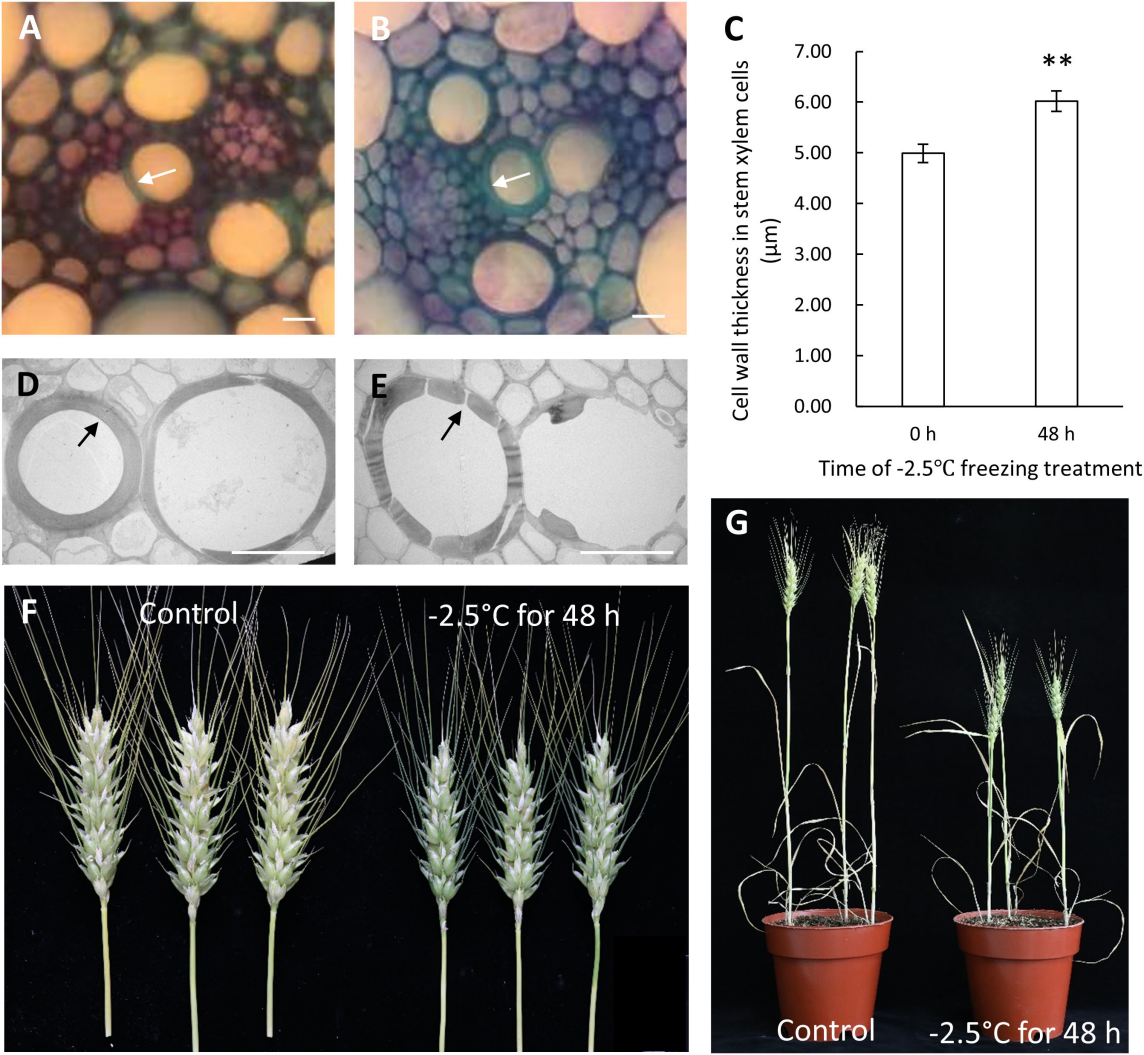
The phenotypic reactions of Zhongmai8444 treated with freezing stress at meiotic stage. **(A)** Morphology of cell walls in xylem cells when plants were grown in normal growth condition. **(B)** Cell wall thickening was observed in plants treated with -2.5°C for 48 h. **(C)** Bar graph represents the cell wall thickness of xylem cells in control and freezing-treated plants. Data are mean ± SE calculated from three biological replicates. ** indicates a significant difference (p-value < 0.01) using a Student’s t-test. **(D)** TEM imaging showed that the cell walls of xylem cells were not disturbed in plants grown in normal growth condition. **(E)** Cell walls of xylem cells were severely damaged when plants were treated with -2.5°C for 48 h and recovered at room temperature. **(F)** Spike length and seed setting were dramatically reduced in plants treated with -2.5°C for 48 h (on the right) compared to plants grown in normal growth condition (on the left). **(G)** Freezing stress at meiotic stage leads to reduced plant height. Scale bars represent 20 μm.

In previous study, we have demonstrated that Zhongmai8444 was extremely sensitive to freezing stress at the meiotic stage/phase by evaluating the damage of freezing on plant stems, leaves, spikes and other organs during the reproductive development (Wang et al., 2022). Consistently, the injury of stems (**Supplementary Figure S3**) and cell wall thickening in the vascular tissue were observed after Zhongmai8444 was treated with -2.5°C for 48 h (**Figures 1A–C**). Cell walls were found to be severely damaged when plants were recovered to normal room temperature (**Figures 1D, E**). In addition, plant height, grain number per spike and seed setting rate were quantified after the plants were treated with freezing stress or grown in normal growth conditions. Results indicated that Zhongmai8444 exhibited significantly reduced height, grain number and seed setting rate after -2.5°C freezing treatment for 48 h at the meiotic stage (**Figures 1F, G**; **Table 1**). To further understand the molecular mechanism underlying wheat frost tolerance, we performed RNA sequencing of the stem to identify differentially expressed genes responsive to freezing stress.

**Table 1:** Effect of 48 h freezing stress (FS) on plant height and seed setting of Zhongmai8444.

	Plant height (cm)	Number of grains per spike	Seed setting rate (%)
Control	43.60 ± 0.78	24.83 ± 1.45	70.63 ± 2.82
48 h FS	31.58 ± 2.41**	18.33 ± 1.05**	56.57 ± 1.83**

Data are mean ± SE calculated from six biological replicates. ** indicates a significant difference (p-value < 0.01) using a Student’s t-test.

### Mapping of RNA-Seq data to wheat genome

Utilizing the paired-end Illumina sequencing technology, the extracted RNA from stem tissue was sequenced to compare gene expression at different time points under freezing stress, samples were named as J1 to J5 accordingly. After quality control, sequencing data yield approximately 70 million high-quality clean reads per library shown in **Supplemental Table S1**. The clean reads shared a GC content of approximately 55% and the alignment rate uniquely mapped to the Chinese Spring iwgsc_refseqv1.0 reference genome (https://urgi.versailles.inra.fr/download/iwgsc/IWGSC_RefSeq_Assemblies/v1.0/) was ranged from 88.96% to 90.76%. In addition, Pearson correlation analysis (PCA) and principal component analysis were performed to determine the quality of biological replications of each sample (**Supplemental Figure S4, Figure S5**). Results showed that the sample J3-2 was relatively dispersed from the other two biological replicates and removed for further analysis. All the other samples had good repeatability with ePearson coefficient higher than 0.87 (**Supplemental Figure S4**).

### Identification of differentially expressed genes under freezing

**Figure 2:**
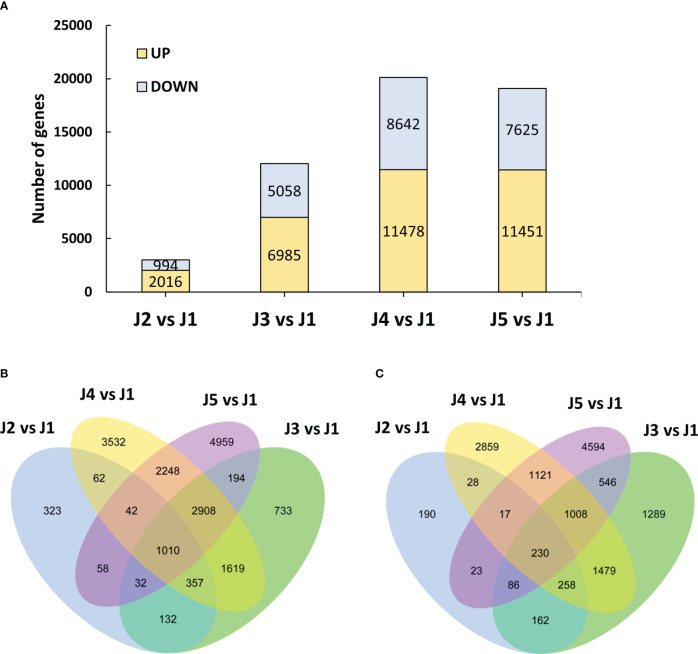
Overview of the transcriptome analysis. **(A)** Differentially expressed genes at each timepoint compared to control plants. **(B)** Venn diagram for the up-regulated genes shared by each time point after -2.5°C freezing treatment. **(C)** Venn diagram for the down-regulated genes shared by each time point after -2.5°C freezing treatment.

To systematically investigate the dynamics of transcriptome profiles of wheat stem, we identified genes that were differentially expressed by at least twofold (|log2FoldChange| > 1 and padj < 0.05) with -2.5°C freezing treatment. A clear difference was observed in the number of up-regulated and repressed DEGs at the indicated time points compared to control plants (**Figure 2A**). A total of 2016 up-regulated and 994 down-regulated genes were detected in stem tissue after 6-h-cooling (J2 vs J1). An increase in the number of significantly up-regulated (5058 to 7000) and down-regulated (6985 to 11778) DEGs was observed with the extended duration of freezing stress (from T2 to T4), which suggested the ongoing adaptation of plants to low temperature conditions. However, there was a small change in the number of DEGs when plants were recovered at 22°C after freezing stress (J5 vs J1). We subsequently constructed the Venn analysis to investigate the number of up-regulated and down-regulated genes that were commonly shared at different time points (**Figures 2B, C** respectively). Results showed that respectively 1010 up-regulated and 230 down-regulated genes were detected common at all time points.

### Gene ontology clustering of DEGs

**Figure 3:**
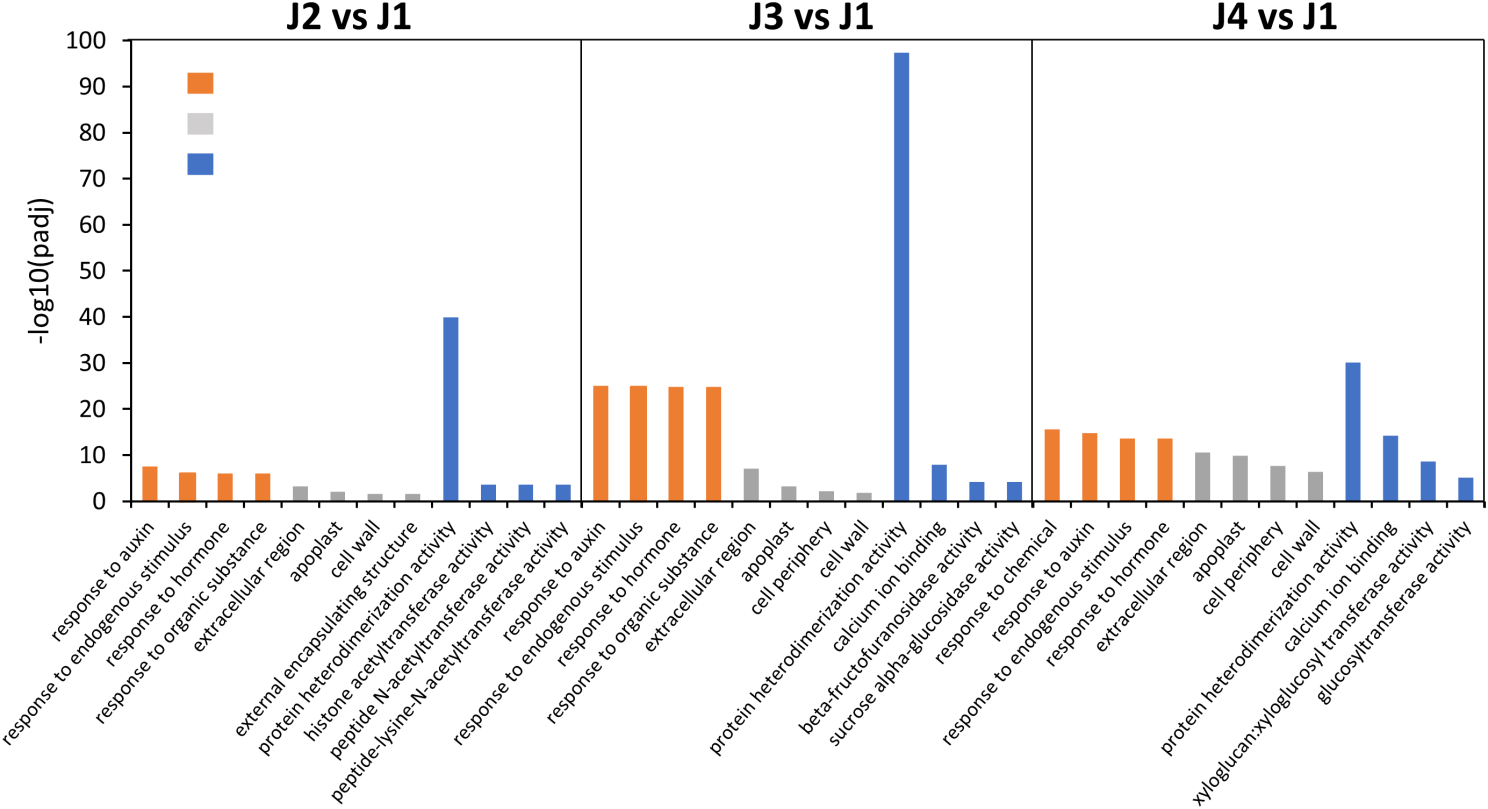
Histogram showing the most significantly enriched GO term from samples collected at each time point during freezing treatment. Color bars represent different functional classifications, including biological process (orange bars), cellular components (grey bars) and molecular function (blue bars).

To functionally characterize the identified DEGs, a GO analysis was performed to classify them into the three categories: molecular function (MF), cellular component (CC) and biological process (BP). Significantly enriched GO terms identified at different time points with freezing treatment (J2 vs J1, J3 vs J1 and J4 vs J1 comparisons) were determined. The top 4 GO terms from each category were respectively presented in **Figure 3** and highly overlap among samples collected from different time points. For instance, in the biological process category, the GO term “response to auxin”, “response to endogenous stimulus”, “response to hormone” were significantly enriched in all samples, suggesting an involvement of genes participated in the signal perception and transduction pathway. Similarly, under the cellular component and molecular function category, the most enriched GO terms “extracellular region”, “apoplast”, “cell wall” and “protein heterodimerization activity” were also detected in all samples. However, the “cell periphery” and “calcium ion binding” were only shared in the samples treated with 24 h or 48 h freezing stress. In addition, in the 48 h freezing treated samples, the other term “xyloglucan:xyloglucosyl transferase activity” and “glucosyltransferase activity” were also related to the cell wall metabolism and consistent with the detected phenotype of cell wall thickening. Therefore, we hypothesized that hormone signal transduction and cell wall biosynthesis process may be modulated to promote plant resistance to freezing.

### Pathways enrichment analysis of DEGs

The KEGG analysis of DEGs in stem was also performed to identify pathways displaying significant changes (padj ≤ 0.05) in response to freezing (**Table 2**). “Plant hormone signal transduction” pathway was significantly enriched at every time point. In addition to this pathway, “circadian rhythm-plant” was also enriched after 6-h-cooling (J2 vs J1). In 24 h freezing treated samples (J3 vs J1), four pathways significantly changed including “plant-pathogen interaction”, “plant hormone signal transduction”, “MAPK signaling pathway-plant”, and “phenylpropanoid biosynthesis”. In addition, analysis revealed that DEGs in 48 h freezing treated samples (J4 vs J1) were associated with “plant-pathogen interaction”, “plant hormone signal transduction”, “phenylpropanoid biosynthesis”, “circadian rhythm-plant”, “photosynthesis-antenna proteins”, “MAPK signaling pathway-plant”, and “starch and sucrose metabolism” pathways. After samples were recovered after freezing treatment (J5 vs J1), the “photosynthesis-antenna proteins”, “photosynthesis”, “plant hormone signal transduction”, and “MAPK signaling pathway-plant” pathways were significantly enriched among DEGs. Consistent with the GO analysis, our results emphasized the involvement of plant hormone in the response of wheat to freezing.

**Table 2:** Significantly enriched gene pathways at each time point following the low temperature treatment.

	Pathway	Gene ratio	padj	KEGG ID
J2 vs J1	Plant hormone signal transduction	16/98	0.000167	dosa04075
	Circadian rhythm-plant	6/98	0.002644	dosa04712
J3 vs J1	Plant-pathogen interaction	42/458	1.01E-05	dosa04626
	Plant hormone signal transduction	38/458	0.002092	dosa04075
	MAPK signaling pathway-plant	23/458	0.005750	dosa04016
	Phenylpropanoid biosynthesis	23/458	0.026783	dosa00940
J4 vs J1	Plant-pathogen interaction	71/797	2.30E-12	dosa04626
	Plant hormone signal transduction	57/797	1.71E-05	dosa04075
	Phenylpropanoid biosynthesis	40/797	2.91E-05	dosa00940
	Circadian rhythm-plant	17/797	3.45E-05	dosa04712
	Photosynthesis-antenna proteins	11/797	8.35E-05	dosa00196
	MAPK signaling pathway-plant	30/797	0.002174	dosa04016
	Starch and sucrose metabolism	33/797	0.002789	dosa00500
J5 vs J1	Photosynthesis-antenna proteins	16/944	6.24E-07	dosa00196
	Photosynthesis	23/944	3.74E-05	dosa00195
	Plant hormone signal transduction	63/944	0.004824	dosa04075
	MAPK signaling pathway-plant	37/944	0.008353	dosa04016

### Representative mostly up-regulated and down-regulated DEGs

Venn analysis identified 1010 up-regulated and 230 down-regulated genes shared by all time points. We listed the expression of top 20 (ranked by the padj value of J4 vs J1 comparison) up-regulated (**Table 3**) and down-regulated DEGs (**Table 4**). We observed several low temperature related genes that have been significantly up regulated, including the abscisic acid (ABA) responsive protein (TraesCS5B01G502200, TraesCS5A01G488500), dehydrin (TraesCS4B01G228000, TraesCS6B01G383200) and late embryogenesis abundant (LEA) proteins (TraesCS1A01G423800, TraesCS3D01G158600, TraesCS3A01G175400). In addition, expression levels of genes involved in diverse biosynthesis process of cell wall, carbohydrates, amino acids, cytoskeleton, and other metabolites were also significantly affected. Few genes encoding the transcription factor were also identified, including those containing GRAM domain, NAC domain, and Myb-like DNA-binding domain. These results indicated that widespread genes showed substantial changes in expression level following freezing temperatures.

**Table 3:** Representative mostly up-regulated genes shared in all time points, shown as log2 value of fold changes.

Gene ID	Description	J2 vs J1	J3 vs J1	J4 vs J1	J5 vs J1
TraesCS5B01G502200	GRAM domain-containing protein/ABA-responsive	4.87	9.72	11.64	10.47
TraesCS6D01G403400	tRNA-2-methylthio-N (6)-dimethylallyladenosine synthase	4.61	7.51	9.66	8.34
TraesCS5B01G069300	HVA22-like protein	2.97	6.53	8.09	5.88
TraesCS7D01G358000	Cysteine-rich/transmembrane domain A-like protein	2.44	7.62	9.21	8.34
TraesCS5D01G481200	NAC domain-containing protein, putative	3.99	7.45	8.52	6.95
TraesCS1A01G423800	Late embryogenesis abundant protein	1.25	6.30	7.69	7.21
TraesCS4B01G228000	Senescence/dehydration-associated protein-like protein	2.72	6.97	7.97	6.84
TraesCS2D01G598500	Acetyl-coenzyme A synthetase	3.47	6.60	8.53	7.51
TraesCS5B01G491500	Actin depolymerizing factor	2.97	5.17	6.61	5.34
TraesCS5A01G488500	GRAM domain-containing protein/ABA-responsive	3.17	6.91	8.95	8.26
TraesCS5D01G491900	Actin depolymerizing factor	2.92	4.93	6.49	4.88
TraesCS1D01G218000	Beta-amylase	5.45	5.85	7.06	4.53
TraesCS3D01G158600	Late embryogenesis abundant protein	6.04	11.19	12.80	12.16
TraesCS3B01G422300	BAG family molecular chaperone regulator 2	2.33	6.54	7.63	6.71
TraesCS5A01G471700	LURP-one-like protein	2.62	5.45	7.12	5.57
TraesCS2A01G295400	Cell wall invertase	2.22	4.98	7.42	7.43
TraesCS5B01G356400	Nudix hydrolase-like protein	1.38	5.48	6.96	4.36
TraesCS6B01G383200	Dehydrin	6.73	12.09	13.96	11.90
TraesCS7A01G065000	Seed maturation protein	5.14	7.86	9.54	7.40
TraesCS3A01G175400	Late embryogenesis abundant protein	4.49	8.29	9.99	8.99

**Table 4:** Representative mostly down-regulated genes common in all time points, shown as log2 value of fold changes.

Gene ID	Description	J2 vs J1	J3 vs J1	J4 vs J1	J5 vs J1
TraesCS2A01G206700	Transmembrane protein	-1.27	-3.32	-5.25	-3.89
TraesCS7D01G078900	Homeobox-leucine zipper family protein	-1.38	-5.56	-6.80	-2.33
TraesCS2B01G233900	Transmembrane protein	-1.18	-2.56	-4.70	-3.65
TraesCS4D01G275000	DUF1677 family protein	-1.43	-4.35	-4.40	-3.66
TraesCS7D01G478900	Cyclin-dependent kinase inhibitor	-1.76	-4.67	-7.28	-3.31
TraesCS5A01G329900	MYB transcription factor	-1.07	-4.20	-4.06	-1.82
TraesCS6A01G401600	Histone H2A	-1.11	-1.00	-2.83	-3.15
TraesCS4A01G030500	Histone H2B	-1.17	-1.55	-4.14	-1.72
TraesCS5A01G534300	Tubulin beta chain	-1.22	-1.89	-3.94	-2.97
TraesCS7B01G432600	Rotundifolia-like protein	-1.23	-5.36	-6.73	-1.15
TraesCS3B01G451100	DUF868 family protein	-1.31	-2.90	-4.17	-3.24
TraesCS6A01G249300	Expansin protein	-1.15	-3.50	-3.72	-3.29
TraesCS5D01G110600	Histone H2A	-1.58	-0.93	-4.79	-1.89
TraesCS7A01G160200	Histidinol dehydrogenase	-1.07	-3.80	-3.99	-3.60
TraesCS1D01G029700	HXXXD-type acyl-transferase family protein	-1.39	-3.56	-6.89	-3.90
TraesCS4A01G096300	Rotundifolia-like protein	-1.80	-2.74	-8.02	-1.17
TraesCS2A01G291400	Unknown protein	-1.46	-2.58	-3.00	-2.70
TraesCS4A01G067900	Glucan endo-1,3-beta-glucosidase, putative	-1.12	-1.91	-3.62	-3.24
TraesCS5D01G222600	Auxin responsive SAUR protein	-2.19	-10.29	-6.27	-2.71
TraesCS7A01G337300	Leucine-rich repeat receptor-like protein kinase	-1.24	-1.71	-2.42	-4.32

### Identification of genes involved in hormone and signal transduction

The GO and KEGG enrichment analyses of the DEGs both highlighted that the plant hormone signal transduction pathway (dosa04075) was particularly affected by the freezing treatment (**Figure 3**; **Table 2**). Among the 1240 DEGs shared by all time points, many genes encoding a protein kinase and probably involved in the signal transduction were detected (**Supplemental Table S2**). More than 40 genes predicted to encode a protein kinase were differentially expressed under freezing treatment. Most of the genes were up regulated while only 6 genes were down regulated by freezing stress. Genes encoding a receptor-like protein kinase constituted the largest group and may play key roles in mediating stress-associated signaling pathway, such as the leucine-rich repeat receptor-like protein kinase (Yang et al., 2022). KEGG analysis of the DEGs suggested that genes encoding protein phosphatase 2C (TraesCS3A01G362200 and TraesCS5A01G183600), which was associated in the carotenoid and ABA biosynthesis, were induced at all time points under freezing condition. The expression of TraesCS3A01G362200 was rapidly up-regulated after 6-h cooling and progressively up-regulated by 5.2 and 10 folds after maintaining at -2.5°C for 24 h and 48 h, respectively, compared to that in control group. The other gene TraesCS5A01G183600 showed similar expression pattern and 8 folds up-regulated expression at time point T4. In addition, 66 DEGs associated with the biosynthesis or signal transduction of the phytohormones like ethylene, auxin, ABA or GA were listed in **Supplemental Table S2**. All the ethylene related genes were induced upon freezing treatment and determined to encode AP2/ERF domain-containing TFs. *ERF* genes are critical for multiple plant species to deal with low temperature stress. In Arabidopsis, *ERF105* has been suggested to operate in conjunction with the well-known CBF regulon (Bolt et al., 2017).

### Identification of transcription factors in response to freezing stress

**Figure 4:**
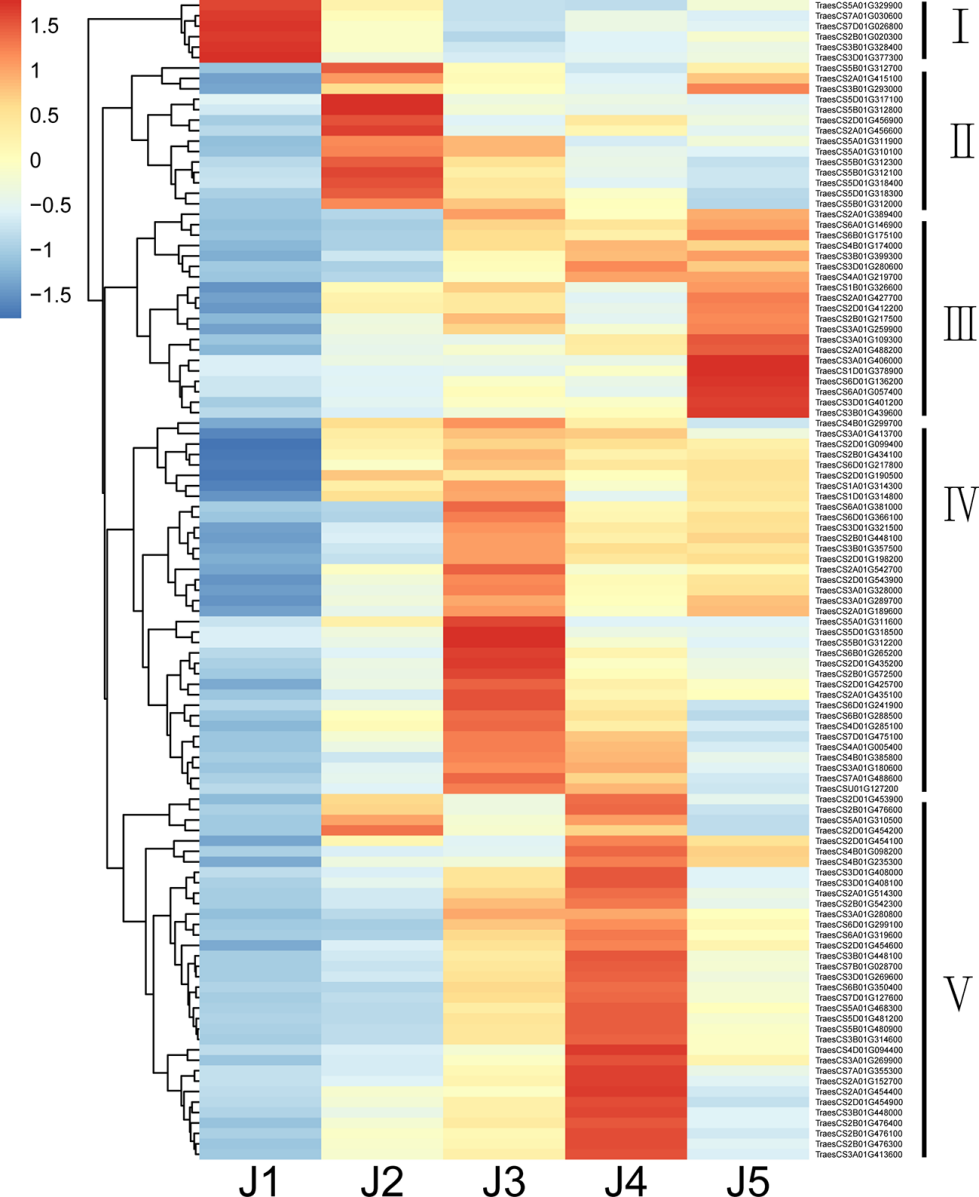
A heat map Showing the expression pattern of differentially expressed TF genes under freezing stress at different time points. Columns and rows in the heat map respectively represent the five time points of freezing treatment and common differentially expressed TFs. Color bars represent scaled FPKM of each TF gene.

Numerous families of TFs are known to play an important role when plants are challenged with various abiotic stresses. In current study, 111 putative TFs were identified to be differentially expressed (|log2FoldChange| > 1 and padj < 0.05) at all time points with freezing treatment (**Table 5**; **Supplemental Table S3**). Genes encode AP2/ERF domain-containing proteins constituted the largest group, followed by zinc finger proteins, NAC domain-containing proteins, MYB TFs, heat shock TFs, WRKY TFs, GRAS TFs, Growth-regulating factors, Homeobox domain TFs, bZIP proteins and ethylene insensitive 3 TF (**Table 5**). The identified TFs were classified into five subgroups based on their expression patterns (**Figure 4**). The expression level of TFs in Subgroup I declined immediately after cooling and became more dramatically reduced after continuous freezing treatment. In contrast, the transcript abundance of TFs in Subgroup II was significantly induced at the early time point and then decreased upon the duration of freezing treatment, which suggests that they play roles in early transduction of the low-temperature signal. Subgroup III TFs were active after freezing but showed a peak of expression when plants were recovered in normal growth condition. Subgroup IV and V, the two largest subgroups, were characterized by genes showing the highest expression after 24-h and 48-h freezing treatment, respectively. In addition, we listed the 10 most highly induced TF genes in stem tissue commonly shared in all time points (**Table 6**). Four genes encoding AP2 domain containing proteins and three NAC domain containing TF genes were identified. The transcript level of two NAC TF encoded genes (TraesCS5D01G481200 and TraesCS5B01G480900) was most significant enhanced subjected to 24-h-freezing which showed the log2 value of fold changes as 8.52 and 7.48, respectively (**Table 6**). These two genes were confirmed to be the homologue of *TaNAC2-5A* which has been reported to play a role in plant freezing tolerance (Mao et al., 2012). Further experiments could be carried out to verify the functional role of other candidate TFs listed in **Table 6**.

**Table 5:** Classification of transcription factor genes differentially expressed at all time points (|log2FoldChange| > 1 and padj < 0.05) upon freezing stress.

Class	Gene number
AP2/ERF domain containing transcription factor	44
Zinc finger transcription factor	19
NAC domain-containing transcription factor	13
MYB transcription factor	13
Heat shock transcription factor	6
WRKY transcription factor	5
GRAS transcription factor	3
Growth-regulating factor	3
Homeobox domain transcription factor	2
bZIP transcription factor	2
Ethylene insensitive 3 transcription factor	1

**Table 6:** Representative mostly up-regulated TFs common at all time points, shown as log2 value of fold changes.

Gene ID	Description	J2 vs J1	J3 vs J1	J4 vs J1	J5 vs J1
TraesCS5D01G481200	NAC domain-containing protein	3.99	7.45	8.52	6.95
TraesCS5B01G480900	NAC domain-containing protein	3.43	6.38	7.48	6.02
TraesCS6A01G146900	WRKY DNA-binding domain	3.95	6.95	7.13	7.50
TraesCS2D01G454900	Zinc-finger of the FCS-type	4.75	5.18	6.61	2.10
TraesCS7D01G127600	Ethylene-responsive transcription factor	2.70	5.28	6.18	4.67
TraesCS7B01G028700	Ethylene-responsive transcription factor	1.86	3.73	4.70	3.20
TraesCS4D01G094400	NAC domain-containing protein, putative	2.04	2.96	5.06	3.43
TraesCS3A01G280800	Heat shock factor	3.53	6.11	6.41	5.54
TraesCS6D01G299100	Ethylene-responsive transcription factor	6.60	12.28	12.90	11.97
TraesCS3D01G321500	Ethylene-responsive factor-like transcription factor	3.71	5.02	4.83	4.95

### Identification of genes associated with cell wall biosynthesis and modification

When subjected to freezing, plants could form a thick cell wall to resist low temperature stress. The GO analysis in our study also indicated that the expression of cell wall related genes was significantly modulated by freezing (**Figure 3**). Therefore, we have selected genes with a putative role in cell wall remodeling shared by J3 vs J1 and J4 vs J1 comparisons (**Table 7**). These genes were participated in the biosynthesis or modification of major cell wall components, such as cellulose, hemicellulose, and pectin. For instance, several genes encoding cellulose synthetic enzymes such as cellulose synthase and cellulose synthase-like proteins, endoglucanases, chitinase-like proteins and cobra-like proteins were identified differentially expressed (**Table 7**). In addition, genes encoding hemicellulose biosynthetic enzyme xyloglucan endo-transglycosylase constituted a large group and have been reported to play an important role in frost tolerance in Arabidopsis (Takahashi D, 2021). Our study indicated that the increased amounts of enzymes involved in cell wall biosynthesis and modification is in accordance with the cell wall thickening observed in wheat stem upon freezing.

**Table 7:** Classification of cell wall related genes differentially expressed (|log2FoldChange| > 1 and padj < 0.05) under 24 h and 48 h freezing treatment.

Gene group	Gene function	Number
Glucan endo-1,3-beta-glucosidase	Beta-1,3-glucan degradation	34
Cellulose synthase and cellulose synthase-like protein	Cell wall biosynthesis	28
Xyloglucan endo-transglycosylase	Xyloglucan metabolism	14
Galactosyltransferase	Galactose biosynthesis	10
Cell wall invertase	Cell wall biosynthesis	10
Endoglucanase	Cellulose crystallization	9
Chitinase-like protein	Cellulose biosynthesis	5
Glucuronoxylan 4-O-methyltransferase-like protein	Xylan biosynthetic process	4
Pectin lyase-like protein	Pectin degradation	3
Cobra-like protein	Cellulose biosynthesis	3
Sucrose synthase	Cell wall biosynthesis	3
Beta-xylosidase	Xylan degradation	2
Alpha-L-arabinofuranosidase	Lignocellulose degradation	1
Endo-1,4-beta-xylanase	Xylan degradation	1
Rhamnogalacturonate lyase	Pectin degradation	1
Callose synthase	Callose biosynthesis	1
Xyloglucan fucosyltransferase	Xyloglucan biosynthesis	1

### Validation of gene expression by real time quantitative PCR analysis

**Figure 5:**
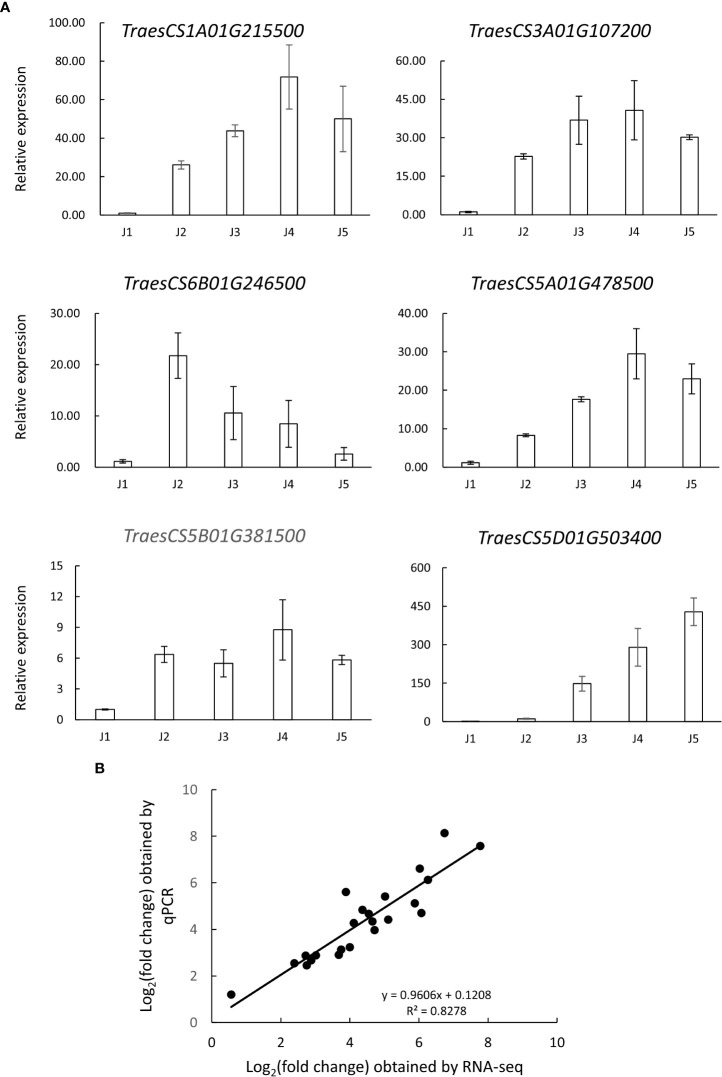
Verification of the expression pattern of selected genes *via* qRT-PCR analysis. **(A)** transcript levels of 6 selected genes in samples collected at different time points (J1-J5). The data are shown as means ± standard errors (SE) in three replicates. **(B)** Correlation analysis of the log2 of gene expression ratios obtained from RNA-seq data and qRT-PCR analysis.

We also performed qRT-PCR analysis to verify the accuracy and reproducibility of transcriptome profiles. According to the literature, six genes potentially involved in low temperature tolerance were selected for the analysis, such as zinc finger proteins (TraesCS6B01G246500 and TraesCS3A01G107200), protein kinase (TraesCS5B01G381500), actin depolymerizing factor (TraesCS5A01G478500), beta-amylase (TraesCS1A01G215500) and ABA-responsive protein (TraesCS5D01G503400) (Peng et al., 2015; Byun et al., 2021; Zhang et al., 2022). Our data showed that the expression pattern of selected genes obtained by qRT-PCR was highly consistent with RNA-Seq data, with the correlation coefficients (R^2^) > 0.8 (**Figure 5**). These results support the reliability of the RNA-Seq analysis.

## Discussion

Freezing injury during the reproductive phase of wheat frequently happens in spring, which has become the limiting factor of wheat production in China. To investigate the effect of freezing stress on wheat growth and reproduction, we performed the freezing treatment on Zhongmai8444 at the meiotic stage in a controlled growth condition. Transcriptomic analysis was subsequently carried out to elucidate the molecular mechanism underlying the frost tolerance of wheat plants.

Consistent with our previous study, Zhongmai8444 showed hypersensitivity to freezing stress at the meiotic stage (Wang et al., 2022). At this time, the developing pollen mother cells were at the meiosis phase and freezing temperature may cause the increase frequency of cellular abnormality of pollen cells. Therefore, wheat plants showed a decrease in seed setting after treated with freezing (**Figure 1F**, **Table 1**). Consistently, it has been reported that Australian wheat cultivars exhibited significant reduction in fertility when subjected to prolonged chilling temperature during meiosis (Barton et al., 2014). The decrease of grain number per spike and 1000-grain weights was observed when winter wheats were exposed to low temperature stress at booting stage (Zhang et al., 2019; Zhang et al., 2021). Results showed that altered sucrose metabolism and inhibited starch biosynthesis in young ear may lead to the reduction of yield. In addition, low temperature stress during floral development is known to cause yield losses in different crop plants such as rice (Li et al., 2022), sorghum (Wood et al., 2006), maize (Tranel et al., 2009), bell pepper (Mercado et al., 1997) and garlic (Shemesh Mayer et al., 2013). Stem injury and reduced plant height were also obvious in freezing-treated plants (**Supplemental Figure S3**, **Figure 1G**, **Table 1**). Vascular tissues in stem play an important role in transporting water, minerals and product of photosynthesis throughout the plants. Injury of stem and vascular tissue would inhibit the transportation of nutrients required for plant growth and development. As a result, wheat plants exhibited reduced height and production.

Cell wall provides the structural integrity as well as the flexibility to a plant cell (Lampugnani et al., 2018). Our findings provide evidence supporting the remodeling of wheat cell wall under freezing stress. Under low temperature conditions, cell wall could help the dehydrated cells maintain the morphology by enhancing its mechanical strength and reduce the rate of cell dehydration by adjusting its structure (Johnson et al., 2018). Previous study demonstrated that chilling stress led to an increase of total cell wall amount as well as structural and compositional changes in cell wall during both cold acclimation and sub-zero acclimation (Takahashi et al., 2019). We identified a range of cell wall associated genes to be dramatically up regulated upon freezing stress by transcriptome analysis (**Table 7**). These genes were implicated in the metabolic events related to major cell wall component biosynthesis and may be critical for plant to resist freezing stress. For instance, Takahashi et al. (2021) suggested a potential role of Arabidopsis xyloglucan endotransglucosylase/hydrolase XTH19 in cell wall remodeling which influenced the freezing tolerance after low temperature acclimation.

To understand the molecular mechanism underlying freezing tolerance, we performed GO and KEGG enrichment analyses on identified DEGs. Our results showed that the plant hormone signal transduction was particularly affected by -2.5°C treatment, suggesting a critical role of phytohormone in conferring response of wheat to the freezing stress. Phytohormones are known to play a significant role in a wide range of adaptive responses to abiotic stresses (Peleg and Blumwald, 2011). When subjected to stresses, accumulation of ABA could promote stomatal closure, enhance water balance, and induce antioxidant defense systems to alleviate oxidative injury (Lee and Luan, 2012). It has been reported that ABA regulated several ICE-CBF pathway-dependent genes, promoting plant’s resistance to freezing (Knight et al., 2004). Furthermore, the involvement of ethylene signaling in low-temperature-stress response has been implicated in several studies (Zhang and Huang, 2010; Shi et al., 2012; Catalá et al., 2014), though its effect is debated. Other hormone such as GA also plays a role in plant responses to low-temperature stress and multiple phytohormones may crosstalk in modulating the expression of cold-responsive genes (Richter et al., 2013).

As the key regulator of transcription, TFs always play a critical role in mediating abiotic stress responses. In current study, we demonstrated that most differentially expressed TF genes belong to AP2/ERF, Zinc finger and NAC family groups (**Table 5** , **Table 6**). The CBF transcription factors belong to the AP2/ERF superfamily and regulate a spectrum of cold-regulated (*COR*) genes. CBFs mediated signaling has been proposed to constitute the predominant cold signaling pathway in many plant species though it has not been well characterized (Park et al., 2015). In wheat, CBF14 and CBF15 TFs were identified to be linked to the frost-tolerance locus Fr-A2 and positively regulate low-temperature-stress responses (Knox et al., 2008; Soltész et al., 2013). Zinc finger and NAC TFs play essential roles in response to various abiotic stresses and have been implicated in the resistance of freezing stress (Hao et al., 2011; Zhang et al., 2016). It has been reported that a C2H2-type zinc finger protein PhZFP1 could regulate cold stress tolerance in Petunia hybrida by modulating galactinol synthesis activity (Zhang et al., 2022). Overexpression of wheat *TaNAC2* gene in Arabidopsis resulted in the enhanced tolerance to multiple abiotic stress (Mao et al., 2012).

Furthermore, based on the listed mostly up-regulated and down-regulated DEGs (**Table 3**; **Table 4**), we proposed several other genes were also participated in freezing responsive process, such as genes encoding dehydrins, actin depolymerizing factors, LEA proteins and beta-amylase. Dehydrins are a distinct group of LEA proteins that exhibited high hydrophilicity and were proved to be an important factor to regulated plant freezing tolerance (Ndong et al., 2002). Overexpression of a dehydrin gene *ShDHN* resulted in the improved freezing tolerance in tomato (Liu et al., 2015). As the best characterized LEA protein, COR15A resided at the membrane surface during dehydration and stabilized cell membranes under freezing stress (Bremer et al., 2017a; Bremer et al., 2017b). In addition, it has been suggested that the actin depolymerizing factor protein may be required for the cytoskeletal arrangement during cold acclimation and important for plants to tolerate freezing (Byun et al., 2021). Our results provide the potential candidates associated with several metabolic pathways involved in freezing responses. Further research is needed to verify the functional roles of these predicted genes and employ them in future breeding program.

**Figure 6:**
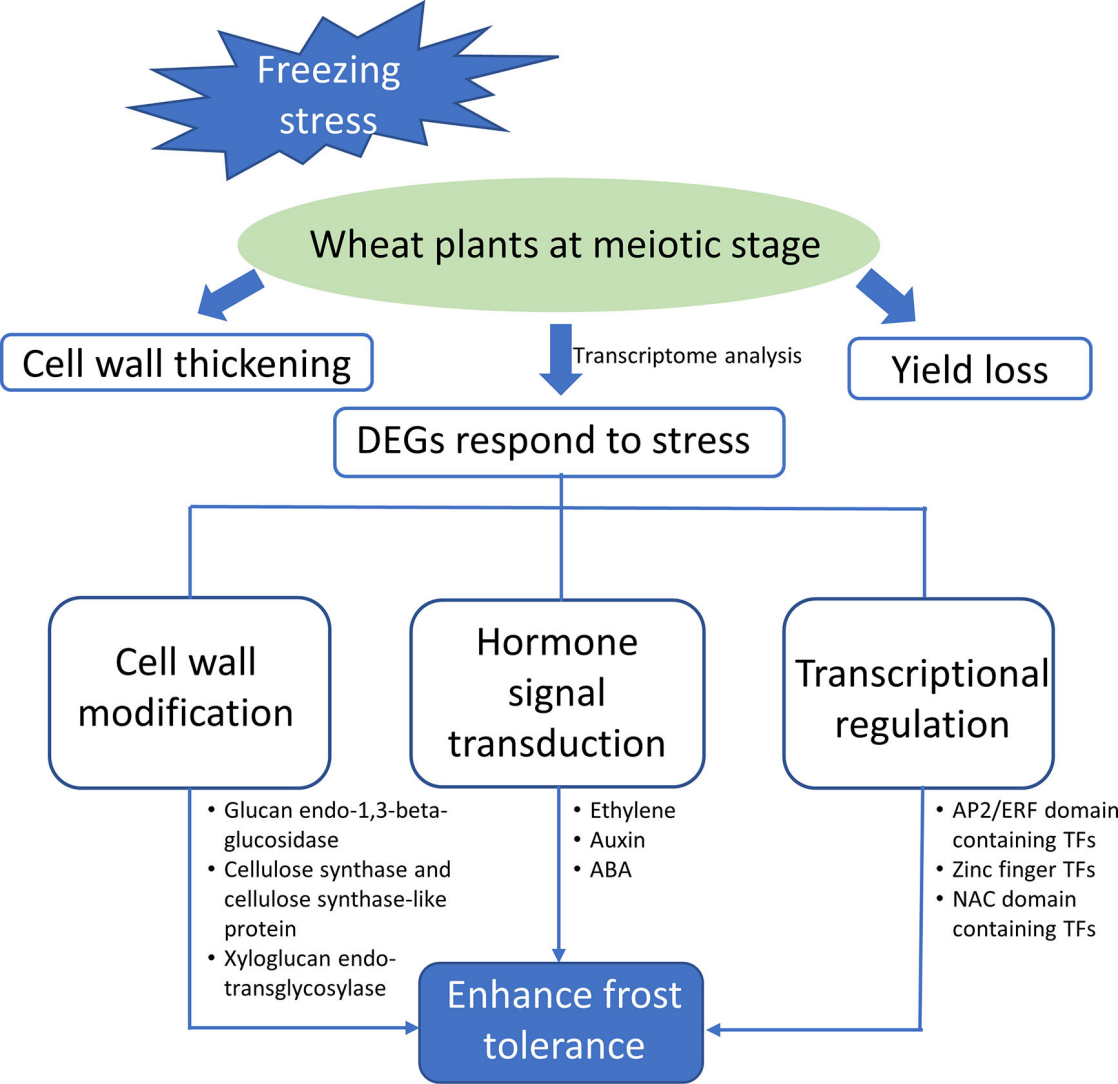
Schematic diagram showing the freezing induced changes of gene expression in wheat stems during meiosis. DEGs were mainly participated in pathways of cell wall modification, hormone signal transduction and transcriptional regulation to promote the freezing tolerance of plants.

In this study, we evaluated the effect of freezing stress on wheat growth and development during meiosis phase. A model for wheat plants respond to freezing is proposed (**Figure 6**). Once subjecting to sub-zero temperature, a series of stress-related genes become active to minimize the damage of freezing and maintain plant’s normal growth, including those encoding TFs, hormone signaling elements, and enzymes associated with cell wall remodeling. The functional validation of these candidate genes will help us better understand the mechanism underlying freezing tolerance and hold great potential for molecular breeding in spring freezing-tolerant wheat varieties.

## Data availability statement

The datasets presented in this study can be found in online repositories. All the raw data have been deposited in the NCBI Sequence Read Archive (SRA) database under BioProject accession number PRJNA901148.

## Author contributions

Conceptualization and supervision, GS, JM, XLiu and YF; methodology, JW and WP; investigation, JW, WP, BZ, XLW, XNW, XYW; bioinformatic analyses, DY and JW; data curation, DY; manuscript preparation, DY, JW and WP; writing-review and editing, GS and DY. All authors contributed to the article and approved the submitted version.

## References

[B1] Barton D. A. Cantrill L. C. Law A. M. Phillips C. G. Sutton B. G. Overall R. L. (2014). Chilling to zero degrees disrupts pollen formation but not meiotic microtubule arrays in T riticum aestivum l. Plant Cell Environ. 37, 2781–2794. doi: 10.1111/pce.12358 24762030

[B2] Bilska-Kos A. Solecka D. Dziewulska A. Ochodzki P. Jończyk M. Bilski H.. (2016). Low temperature caused modifications in the arrangement of cell wall pectins due to changes of osmotic potential of cells of maize. Protoplasma 254, 713–724. doi: 10.1007/s00709-016-0982-y 27193139 PMC5309300

[B3] Bolt S. Zuther E. Zintl S. Hincha D. K. Schmülling T. (2017). ERF105 is a transcription factor gene of arabidopsis thaliana required for freezing tolerance and cold acclimation. Plant Cell Environ. 40, 108–120. doi: 10.1111/pce.12838 27723941

[B4] Bremer A. Kent B. Hauß T. Thalhammer A. Yepuri N. R. Darwish T. A.. (2017a). Intrinsically disordered stress protein COR15A resides at the membrane surface during dehydration. Biophys. J. 113, 572–579. doi: 10.1016/j.bpj.2017.06.027 28793212 PMC5549682

[B5] Bremer A. Wolff M. Thalhammer A. Hincha D. K. (2017b). Folding of intrinsically disordered plant LEA proteins is driven by glycerol-induced crowding and the presence of membranes. FEBS J. 284, 919–936. doi: 10.1111/febs.14023 28109185

[B6] Byun M. Y. Cui L. H. Lee A. Oh H. G. Yoo Y.-H. Lee J.. (2021). Abiotic stress-induced actin-depolymerizing factor 3 from deschampsia antarctica enhanced cold tolerance when constitutively expressed in rice. Front. Plant Sci. 12. doi: 10.3389/fpls.2021.734500 PMC8506025 34650582

[B7] Catalá R. López-Cobollo R. Mar Castellano M. Angosto T. Alonso J. M. Ecker J. R.. (2014). The arabidopsis 14-3-3 protein RARE COLD INDUCIBLE 1A links low-temperature response and ethylene biosynthesis to regulate freezing tolerance and cold acclimation. Plant Cell 26, 3326–3342. doi: 10.1105/tpc.114.127605 25122152 PMC4371832

[B8] Chen X. Lin T. Lin F. Zhang Y. Su H. Hu Y.. (2020). Research progress on damage mechanism and prevention and control measures of late spring coldness of wheat in huanghuai region. J. Triticeae Crops 40, 243–250. doi: 10.7606/ji.ssn.1009-1041.2020.02.14. (In Chinese with English abstract).

[B9] Ding Y. Shi Y. Yang S. (2019). Advances and challenges in uncovering cold tolerance regulatory mechanisms in plants. New Phytol. 222, 1690–1704. doi: 10.1111/nph.15696 30664232

[B10] Draeger T. Moore G. (2017). Short periods of high temperature during meiosis prevent normal meiotic progression and reduce grain number in hexaploid wheat (*Triticum aestivum l.*). Theor. Appl. Genet. 130, 1785–1800. doi: 10.1007/s00122-017-2925-1 28550436 PMC5565671

[B11] Frederiks T. M. Christopher J. T. Sutherland M. W. Borrell A. K. (2015). Post-head-emergence frost in wheat and barley: Defining the problem, assessing the damage, and identifying resistance. J. Exp. Bot. 66, 3487–3498. doi: 10.1093/jxb/erv088 25873656

[B12] Guo W. L. Chen R. G. Gong Z. H. Yin Y. X. Ahmed S. S. He Y. M. (2012). Exogenous abscisic acid increases antioxidant enzymes and related gene expression in pepper (Capsicum annuum) leaves subjected to chilling stress. Genet. Mol. Res. 11, 4063–4080. doi: 10.4238/2012.September.10.5 23079969

[B13] Hao Y. J. Wei W. Song Q. X. Chen H. W. Zhang Y. Q. Wang F.. (2011). Soybean NAC transcription factors promote abiotic stress tolerance and lateral root formation in transgenic plants. Plant J. 68, 302–313. doi: 10.1111/j.1365-313X.2011.04687.x 21707801

[B14] Huang J. Zhao X. Chory J. (2019). The arabidopsis transcriptome responds specifically and dynamically to high light stress. Cell Rep. 29, 4186–4199, e4183. doi: 10.1016/j.celrep.2019.11.051 31851942 PMC7030938

[B15] Hu Y. Jiang Y. Han X. Wang H. Pan J. Yu D. (2017). Jasmonate regulates leaf senescence and tolerance to cold stress: crosstalk with other phytohormones. J. Exp. Bot. 68, 1361–1369. doi: 10.1093/jxb/erx004 28201612

[B16] Hu Y. Jiang L. Wang F. Yu D. (2013). Jasmonate regulates the inducer of CBF expression-c-repeat binding factor/DRE binding factor1 cascade and freezing tolerance in arabidopsis. Plant Cell 25, 2907–2924. doi: 10.1105/tpc.113.112631 23933884 PMC3784588

[B17] Jian C. Zeng B. Zhang M. Shaojun X. Wang G. (2014). Dynamic transcriptome landscape of maize embryo and endosperm development. Plant Physiol. 166, 252–264. doi: 10.1104/pp.114.240689 25037214 PMC4149711

[B18] Johnson K. L. Gidley M. J. Bacic A. Doblin M. S. (2018). Cell wall biomechanics: a tractable challenge in manipulating plant cell walls 'fit for purpose'! Curr. Opin. Biotechnol. 49, 163–171. doi: 10.1016/j.copbio.2017.08.013 28915438

[B19] Kim T. E. Kim S. K. Han T. J. Lee J. S. Chang S. C. (2002). ABA and polyamines act independently in primary leaves of cold-stressed tomato (Lycopersicon esculentum). Physiol. Plant 115, 370–376. doi: 10.1034/j.1399-3054.2002.1150306.x 12081529

[B20] Kim D. Langmead B. Salzberg S. L. (2015). HISAT: A fast spliced aligner with low memory requirements. Nat. Methods 12, 357–360. doi: 10.1038/nmeth.3317 25751142 PMC4655817

[B21] Knight H. Zarka D. G. Okamoto H. Thomashow M. F. Knight M. R. (2004). Abscisic acid induces CBF gene transcription and subsequent induction of cold-regulated genes *via* the CRT promoter element. Plant Physiol. 135, 1710–1717. doi: 10.1104/pp.104.043562 15247382 PMC519084

[B22] Knox A. K. Li C. Vágújfalvi A. Galiba G. Stockinger E. J. Dubcovsky J. (2008). Identification of candidate CBF genes for the frost tolerance locus fr-a m 2 in triticum monococcum. Plant Mol. Biol. 67, 257–270. doi: 10.1007/s11103-008-9316-6 18317935

[B23] Lampugnani E. R. Khan G. A. Somssich M. Persson S. (2018). Building a plant cell wall at a glance. J. Cell. Sci. 131, jcs207373. doi: 10.1242/jcs.207373 29378834

[B24] Le M. Q. Pagter M. Hincha D. K. (2015). Global changes in gene expression, assayed by microarray hybridization and quantitative RT-PCR, during acclimation of three arabidopsis thaliana accessions to sub-zero temperatures after cold acclimation. Plant Mol. Biol. 87, 1. doi: 10.1007/s11103-014-0256-z 25311197

[B25] Lee S. C. Luan S. (2012). ABA signal transduction at the crossroad of biotic and abiotic stress responses. Plant Cell Environ. 35, 53–60. doi: 10.1111/j.1365-3040.2011.02426.x 21923759

[B26] Liu F. Wan Y. Cao W. Zhang Q. Li Y. Li Y.. (2021). Advances on identification of wheat cold tolerance in spring. J. Plant Genet. Res. 22, 1193–1199. doi: 10.13430/j.cnki.jpgr.20210113001

[B27] Liu H. Yu C. Li H. Ouyang B. Wang T. Zhang J.. (2015). Overexpression of ShDHN, a dehydrin gene from solanum habrochaites enhances tolerance to multiple abiotic stresses in tomato. Plant Sci. 231, 198–211. doi: 10.1016/j.plantsci.2014.12.006 25576005

[B28] Li J. Zhang Z. Chong K. Xu Y. (2022). Chilling tolerance in rice: Past and present. J. Plant Physiol. 268, 153576. doi: 10.1016/j.jplph.2021.153576 34875419

[B29] Love M. I. Huber W. Anders S. (2014). Moderated estimation of fold change and dispersion for RNA-seq data with DESeq2. Genome Biol. 15, 550. doi: 10.1186/s13059-014-0550-8 25516281 PMC4302049

[B30] Mao X. Zhang H. Qian X. Li A. Zhao G. Jing R. (2012). TaNAC2, a NAC-type wheat transcription factor conferring enhanced multiple abiotic stress tolerances in arabidopsis. J. Exp. Bot. 63, 2933–2946. doi: 10.1093/jxb/err462 22330896 PMC3350912

[B31] Mercado J. Mar Trigo M. Reid M. Valpuesta V. Quesada M. (1997). Effects of low temperature on pepper pollen morphology and fertility: evidence of cold induced exine alterations. J. Horti. Sci. 72, 317–326. doi: 10.1080/14620316.1997.11515518

[B32] Nakabayashi R. Saito K. (2015). Integrated metabolomics for abiotic stress responses in plants. Curr. Opin. Plant Biol. 24, 10–16. doi: 10.1016/j.pbi.2015.01.003 25618839

[B33] Ndong C. Danyluk J. Wilson K. E. Pocock T. Huner N. P. Sarhan F. (2002). Cold-regulated cereal chloroplast late embryogenesis abundant-like proteins. molecular characterization and functional analyses. Plant Physiol. 129, 1368–1381. doi: 10.1104/pp.001925 12114590 PMC166530

[B34] Ou X. Wang Y. (2019). Preliminary study on wheat breading for late spring coldness tolerance in south of huanghuai region. J. Trit. Crops 39, 560–566. doi: 10.7606/j.issn.1009-1041.2019.05.07

[B35] Panter P. E. Kent O. Dale M. Smith S. J. Skipsey M. Thorlby G.. (2019). MUR1-mediated cell-wall fucosylation is required for freezing tolerance in arabidopsis thaliana. New Phytol.t 224, 1518–1531. doi: 10.1111/nph.16209 PMC6899859 31549420

[B36] Park S. Lee C. M. Doherty C. J. Gilmour S. J. Kim Y. Thomashow M. F. (2015). Regulation of the arabidopsis CBF regulon by a complex low-temperature regulatory network. Plant J. 82, 193–207. doi: 10.1111/tpj.12796 25736223

[B37] Parrotta L. Faleri C. Guerriero G. Cai G. (2019). Cold stress affects cell wall deposition and growth pattern in tobacco pollen tubes. Plant Sci. 283, 329–342. doi: 10.1016/j.plantsci.2019.03.010 31128704

[B38] Peleg Z. Blumwald E. (2011). Hormone balance and abiotic stress tolerance in crop plants. Curr. Opin. Plant Biol. 14, 290–295. doi: 10.1016/j.pbi.2011.02.001 21377404

[B39] Peng T. Zhu X. Duan N. Liu J. H. (2015). PtrBAM1, a β-amylase-coding gene of poncirus trifoliata, is a CBF regulon member with function in cold tolerance by modulating soluble sugar levels. Plant Cell Environ. 37, 2754–2767. doi: 10.1111/pce.12384 24905016

[B40] Richter R. Bastakis E. Schwechheimer C. (2013). Cross-repressive interactions between SOC1 and the GATAs GNC and GNL/CGA1 in the control of greening, cold tolerance, and flowering time in arabidopsis. Plant Physiol. 162, 1992–2004. doi: 10.1104/pp.113.219238 23739688 PMC3729777

[B41] Sga B. Quan X. A. Dm A. Wz A. Zx A. Mz A.. (2019). Transcriptomics profiling in response to cold stress in cultivated rice and weedy rice. Gene 685, 96–105. doi: 10.1016/j.gene.2018.10.066 30389557

[B42] Sharabi-Schwager M. Samach A. Porat R. (2010). Overexpression of the CBF2 transcriptional activator in arabidopsis suppresses the responsiveness of leaf tissue to the stress hormone ethylene. Plant Biol. 12, 630–638. doi: 10.1111/j.1438-8677.2009.00255.x 20636906

[B43] Shemesh Mayer E. Winiarczyk K. Błaszczyk L. Kosmala A. Rabinowitch H. D. Kamenetsky R. (2013). Male Gametogenesis and sterility in garlic (Allium sativum l.): barriers on the way to fertilization and seed production. Planta 237, 103–120. doi: 10.1007/s00425-012-1748-1 22986686

[B44] Shi Y. Tian S. Hou L. Huang X. Zhang X. Guo H.. (2012). Ethylene signaling negatively regulates freezing tolerance by repressing expression of CBF and type-a ARR genes in arabidopsis. Plant Cell 24, 2578–2595. doi: 10.1105/tpc.112.098640 22706288 PMC3406918

[B45] Soltész A. Smedley M. Vashegyi I. Galiba G. Harwood W. Vágújfalvi A. (2013). Transgenic barley lines prove the involvement of TaCBF14 and TaCBF15 in the cold acclimation process and in frost tolerance. J. Exp. Bot. 64, 1849–1862. doi: 10.1093/jxb/ert050 23567863 PMC3638819

[B46] Sugiyama S. Shimazaki T. (2007). Increased cell-wall mass and resistance to freezing and snow mold during cold acclimation of winter wheat under field conditions. Plant Prod. Sci. 10, 383–390. doi: 10.1626/pps.10.383

[B47] Su C. F. Wang Y. C. Hsieh T. H. Lu C. A. Yu T. (2010). A novel MYBS3-dependent pathway confers cold tolerance in rice. Plant Physiol. 153, 145–158. doi: 10.1104/pp.110.153015 20130099 PMC2862423

[B48] Takahashi D. Gorka M. Erban A. Graf A. Kopka J. Zuther E.. (2019). Both cold and sub-zero acclimation induce cell wall modification and changes in the extracellular proteome in arabidopsis thaliana. Sci. Rep. 9, 1–15. doi: 10.1038/s41598-019-38688-3 30783145 PMC6381082

[B49] Takahashi J. K. Hao P. Tuong T. Erban A Sampathkumar A Bacic A. (2021). Cell wall modification by the xyloglucan endotransglucosylase/hydrolase XTH19 influences freezing tolerance after cold and sub-zero acclimation. Plant Cell Environ. 44, 915–930. doi: 10.1111/pce.13953 33190295

[B50] Tranel D. Knapp A. Perdomo A. (2009). Chilling effects during maize tassel development and the lack of compensational plasticity. Crop Sci. 49, 1852–1858. doi: 10.1111/pce.13953

[B51] Vigani G. Bohic S. Faoro F. Vekemans B. Vincze L. Terzano R. (2018). Cellular fractionation and nanoscopic X-ray fluorescence imaging analyses reveal changes of zinc distribution in leaf cells of iron-deficient plants. Front. Plant Sci. 9, 1112. doi: 10.3389/fpls.2018.01112 30123229 PMC6085429

[B52] Wang J. Liu Y. Yao D. Zou J. Xiao S. Sun G. (2022). Identification on sensitivity of wheat to low temperature at reproductive stages. Crop J. 48, 1721–1729. doi: 10.3724/SP.J.1006.2022.11045.

[B53] Wang B. Liu C. Zhang D. He C. Li Z. (2019). Effects of maize organ-specific drought stress response on yields from transcriptome analysis. BMC Plant Biol. 19, 335. doi: 10.1186/s12870-019-1941-5 31370805 PMC6676540

[B54] Wang X. Wu D. Yang Q. Zeng J. Jin G. Chen Z.-H.. (2016). Identification of mild freezing shock response pathways in barley based on transcriptome profiling. Front. Plant Sci. 7. doi: 10.3389/fpls.2016.00106 PMC4744895 26904070

[B55] Willick I. R. Takahashi D. Fowler D. B. Uemura M. Tanino K. K. (2017). Tissue-specific changes in apoplastic proteins and cell wall structure during cold acclimation of winter wheat crowns. J. Exp. Bot. 10, 1221–1234. doi: 10.1093/jxb/erx450 PMC6019019 29373702

[B56] Wood A. Tan D. Mamun E. Sutton B. (2006). Sorghum can compensate for chilling-induced grain loss. J. Agron. Crop Sci. 192, 445–451. doi: 10.1111/j.1439-037X.2006.00233.x

[B57] Yang L. Gao C. Jiang L. (2022). Leucine-rich repeat receptor-like protein kinase AtORPK1 promotes oxidative stress resistance in an AtORPK1-AtKAPP mediated module in arabidopsis. Plant Sci. 315, 111147. doi: 10.1016/j.plantsci.2021.111147 35067310

[B58] Yang L. Smyth G. K. Wei S. (2014). featureCounts: an efficient general purpose program for assigning sequence reads to genomic features. Bioinformatics 30, 923–930. doi: 10.1093/bioinformatics/btt656 24227677

[B59] Yang Y. Yao N. Jia Z. Duan J. Chen F. Sang Z.. (2016). Effect of exogenous abscisic acid on cold acclimation in two magnolia species. Biol. Plant 60, 555–562. doi: 10.1007/s10535-016-0623-5

[B60] Yu J. Cang J. Lu Q. Fan B. Xu Q. Li W.. (2020). ABA enhanced cold tolerance of wheat ‘dn1’via increasing ROS scavenging system. Plant Signal Behav. 15, 1780403. doi: 10.1080/15592324.2020.1780403 32619128 PMC8570709

[B61] Yu G. Wang L. G. Han Y. He Q. Y. (2012). clusterProfiler: An r package for comparing biological themes among gene clusters. Omics 16, 284–287. doi: 10.1089/omi.2011.0118 22455463 PMC3339379

[B62] Zadoks J. C. Chang T. T. Konzak C. F. (1974). A decimal code for the growth stages of cereals. Weed Res. 14, 415–421. doi: 10.1111/j.1365-3180.1974.tb01084.x

[B63] Zhang Z. Huang R. (2010). Enhanced tolerance to freezing in tobacco and tomato overexpressing transcription factor TERF2/LeERF2 is modulated by ethylene biosynthesis. Plant Mol. Biol. 73, 241–249. doi: 10.1007/s11103-010-9609-4 20135196

[B64] Zhang H. Sun Z. Feng S. Zhang J. Zhang F. Wang W.. (2022). The C2H2-type zinc finger protein PhZFP1 regulates cold stress tolerance by modulating galactinol synthesis in petunia hybrida. J. Exp. Bot. 73, 6434–6448. doi: 10.1093/jxb/erac274 35726094

[B65] Zhang W. Wang J. Huang Z. Mi L. Xu K. Wu J.. (2019). Effects of low temperature at booting stage on sucrose metabolism and endogenous hormone contents in winter wheat spikelet. Front. Plant Sci. 10. doi: 10.3389/fpls.2019.00498 PMC6482243 31057594

[B66] Zhang L. Zhang L. Xia C. Zhao G. Jia J. Kong X. (2016). The novel wheat transcription factor TaNAC47 enhances multiple abiotic stress tolerances in transgenic plants. Front. Plant Sci. 6 1174. doi: 10.3389/fpls.2015.01174 26834757 PMC4716647

[B67] Zhang W. Zhao Y. Li L. Xu X. Yang L. Luo Z.. (2021). The effects of short-term exposure to low temperatures during the booting stage on starch synthesis and yields in wheat grain. Front. Plant Sci. 12. doi: 10.3389/fpls.2021.684784 PMC8300962 34305982

[B68] Zhao Y. Zhou M. Xu K. Li J. Li S. Zhang S.. (2019). Integrated transcriptomics and metabolomics analyses provide insights into cold stress response in wheat. Crop J. 7, 857–866. doi: 10.1016/j.cj.2019.09.002

