# Supplementary material for: Transcriptomic profiling of wheat stem during meiosis in response to freezing stress
Source: Front Plant Sci. 2023 Jan 12;13:1099677. doi: 10.3389/fpls.2022.1099677 (PMC9878610; doi:10.3389/fpls.2022.1099677)
Supplement: Supplementary file 2 [file DataSheet_1.docx]

Supplementary Materials


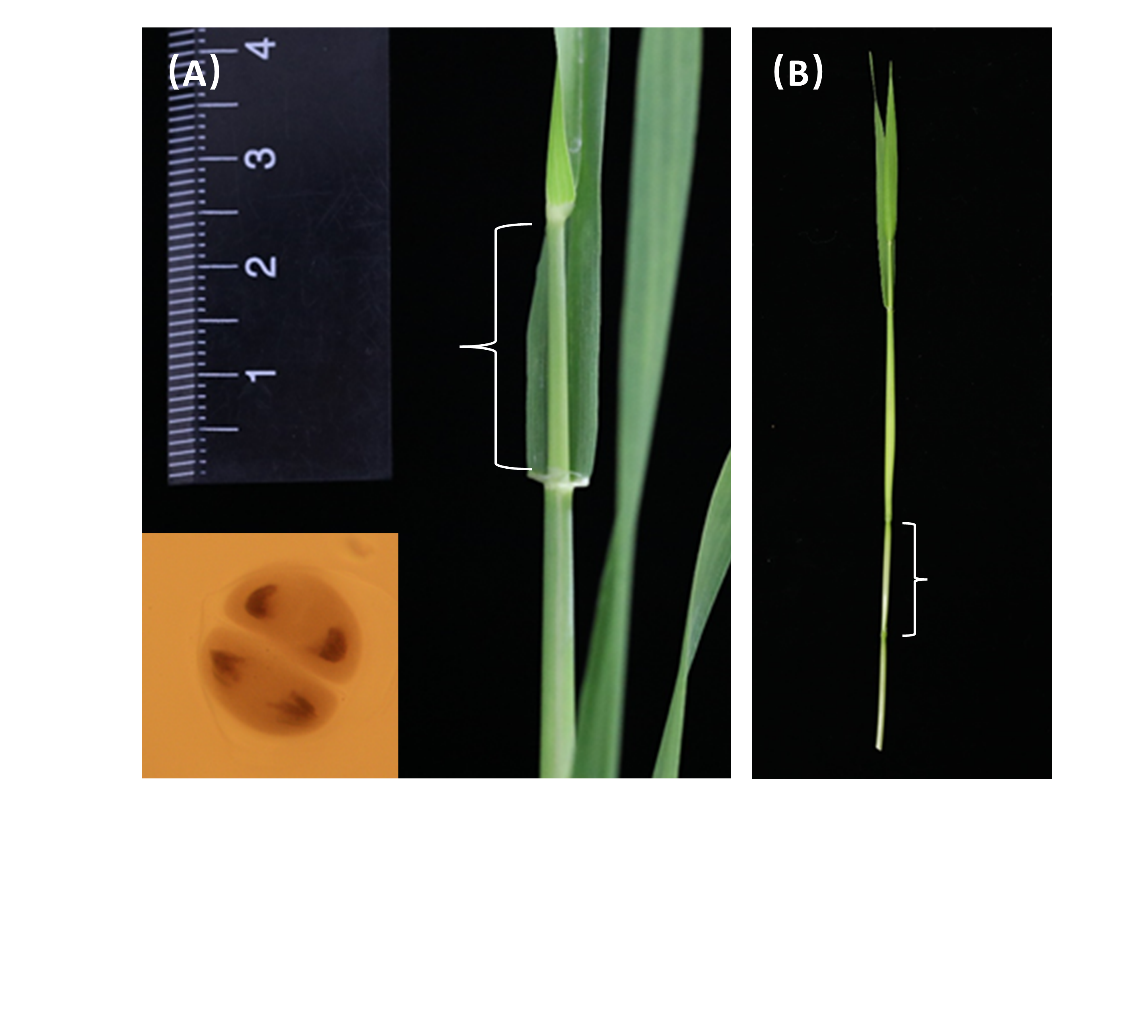


**Figure S1.** Sample collection for transcriptome analysis. (A) When the pollen mother cells of immature anther were at the meiotic stage, the distance from flag leaf to the next leaf of Zhongmai8444 was about 2 to 3 cm. (B) The second internode of stem tissues were collected for transcriptome analysis.

Figure S2. Different time points for sample collection.


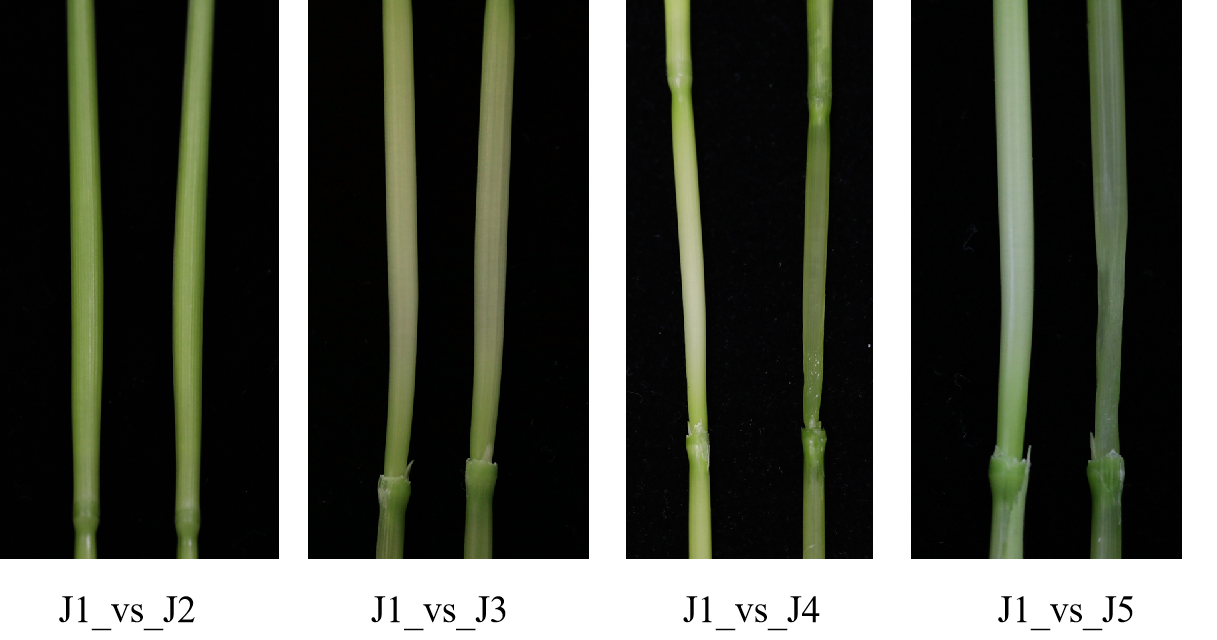


**J1 vs J3**

**J1 vs J4**

**J1 vs J5**

**J1 vs J2**

**Figure S3.** Morphology of stem tissue at different time points. Stems on the left of each picture are grown in normal condition and set as control. Stems on the right of picture were treated with freezing.


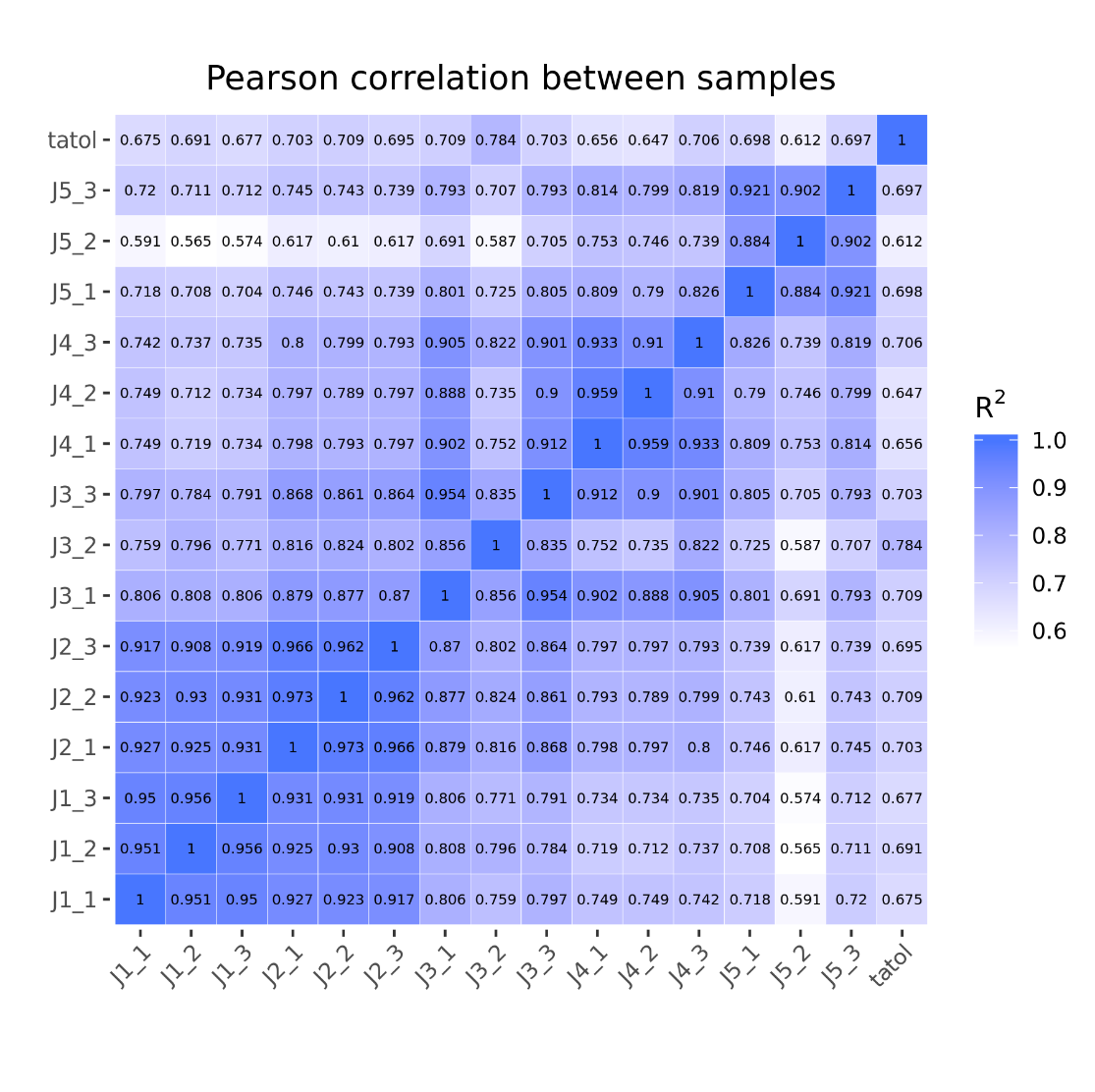


**Figure S4.** Heat map showing the correlation between samples.


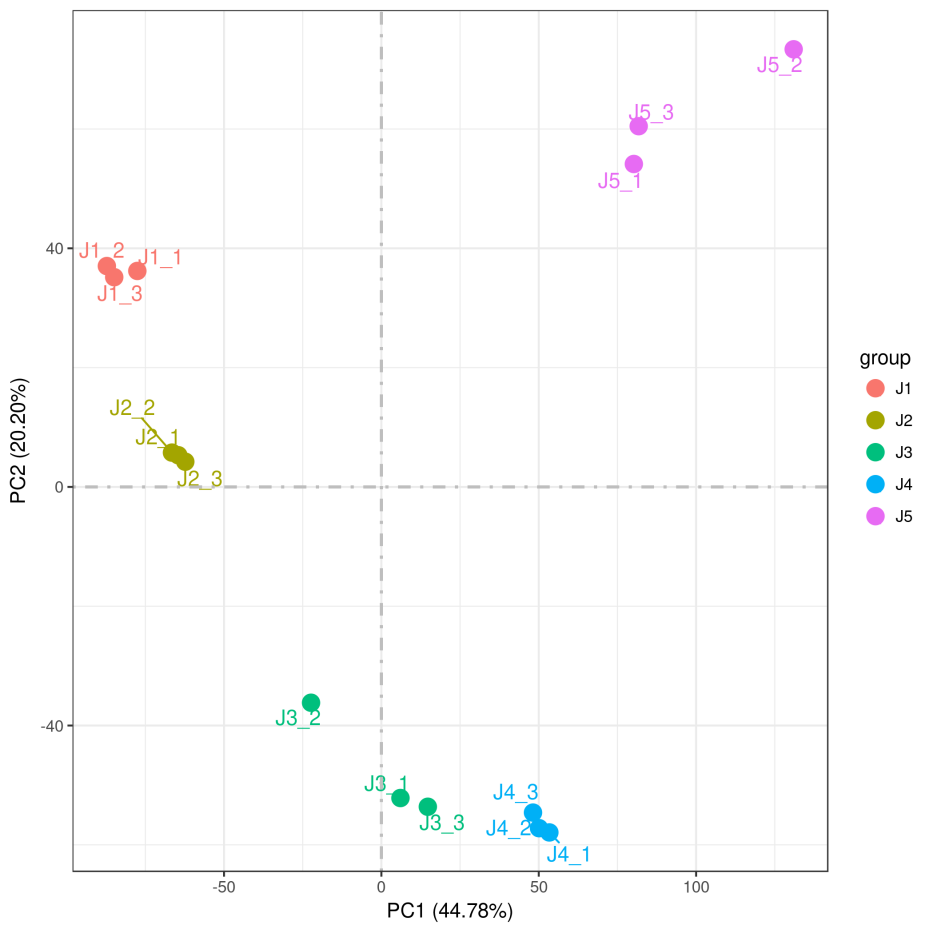


**Figure S5.** Principal component analysis showing the quality of biological replications of each sample.
